# Epigenetic Silencing of miR-9 Promotes Migration and Invasion by EZH2 in Glioblastoma Cells

**DOI:** 10.3390/cancers12071781

**Published:** 2020-07-03

**Authors:** Yi-Chung Chien, Jia-Ni Chen, Ya-Huey Chen, Ruey-Hwang Chou, Han-Chung Lee, Yung-Luen Yu

**Affiliations:** 1 Graduate Institute of Biomedical Sciences, China Medical University, Taichung 404, Taiwan; hardway19800710@gmail.com (Y.-C.C.); nicolejiani@gmail.com (J.-N.C.); yahuey@mail.cmu.edu.tw (Y.-H.C.); rhchou@mail.cmu.edu.tw (R.-H.C.); 2 Center for Molecular Medicine, China Medical University Hospital, Taichung 404, Taiwan; 3 Drug Development Center, China Medical University, Taichung 404, Taiwan; 4 Department of Biotechnology, Asia University, Taichung 413, Taiwan; 5 School of Medicine, College of Medicine, China Medical University, Taichung 404, Taiwan; 6 Department of Neurosurgery, China Medical University Hospital, Taichung 404, Taiwan

**Keywords:** enhancer of zeste homolog 2, CXCR4, miR-9, glioblastoma, invasion

## Abstract

Glioblastoma (GBM) is the most common primary brain tumor in adults. Tumor invasion is the major reason for treatment failure and poor prognosis in GBM. Inhibiting migration and invasion has become an important therapeutic strategy for GBM treatment. Enhancer of zeste homolog 2 (EZH2) and C-X-C motif chemokine receptor 4 (CXCR4) have been determined to have important roles in the occurrence and development of tumors, but the specific relationship between EZH2 and CXCR4 expression in GBM is less well characterized. In this study, we report that *EZH2* and *CXCR4* were overexpressed in glioma patients. Furthermore, elevated *EZH2* and *CXCR4* were correlated with shorter disease-free survival. In three human GBM cell lines, EZH2 modulated the expression of miR-9, which directly targeted the oncogenic signaling of CXCR4 in GBM. The ectopic expression of miR-9 dramatically inhibited the migratory capacity of GBM cells in vitro. Taken together, our results indicate that miR-9, functioning as a tumor-suppressive miRNA in GBM, is suppressed through epigenetic silencing by EZH2. Thus, miR-9 may be an attractive target for therapeutic intervention in GBM.

## 1. Introduction

Glioblastoma (GBM) remains among the most devastating cancers, with a median survival of less than 15 months and virtually no survival beyond 5 years [1]. GBM is the most aggressive form of human brain tumors; its poor prognosis derives largely from the high degree of invasiveness of these cells throughout the brain parenchyma, which is the leading cause of the resistance of GBM to radiation therapy and chemotherapy [2,3]. GBM cells are highly proliferative and are notorious because of their capacity to induce angiogenic blood vessel sprouting [4].

Enhancer of zeste homolog 2 (EZH2), which catalyzes the trimethylation of histone H3 lysine 27 (H3K27me3), is a histone methyltransferase [5]. Polycomb-repressor complex 2 (PRC2) comprises EZH2, SUZ12 polycomb repressive complex 2 subunit (SUZ12), and embryonic ectoderm development (EED); PRC2 functions as a transcriptional repressor and plays an important role in coordinating gene expression and repression during many developmental processes [6]. Recently, various oncogenic properties have been linked to EZH2, including impaired cellular differentiation, enhanced proliferation, and tumor growth [7,8,9,10]. EZH2 is overexpressed in various cancers and is associated with decreased patient survival [7,8,9,10,11,12,13]. Epigenetic aberrations have been recently implicated in the development of GBM [14,15]. For example, in GBM, the cancer-specific concordant hyper-methylation of the CpG islands suppresses the promoters of hundreds of genes, giving rise to the ‘‘glioma-CpG island methylator phenotype (G-CIMP)’’ [15]. In addition to DNA methylation, another epigenetic mechanism involved in carcinogenesis appears to involve the malfunction or dysregulation of proteins that function in chromatin modification and remodeling. It had been reported that high levels of EZH2 expression are associated with the invasion and metastasis of malignant tumors, such as breast cancers and non-small cell lung cancers [13,16]. Recently, some reports have also described that H3K27me3 by EZH2 is not always associated with promoter DNA methylation, which silences EZH2 target genes [17,18,19,20]. Yin Yang 1 (YY1), a dual function transcription factor, is known to regulate the transcriptional activation and repression of many genes that are involved in various homeostatic processes and diseases including cancer [21,22]. EZH2 could be recruited to the target gene/miRNA promoters via the DNA-binding protein YY1 and mediate histone H3 lysine 27 trimethylation to silence the expression of genes/miRNAs involved in skeletal muscle differentiation and hepatocellular carcinoma progression [23,24].

The epigenetic regulation of tumor-suppressive microRNAs (miRNAs) has emerged as a critical signaling pathway involved in tumorigenesis. miRNAs are a family of endogenous small non-coding RNAs that play key roles in post-transcriptional gene regulation through targeting protein-encoding mRNAs [25,26]. EZH2 can directly inhibit the miR-29, miR-181, and miR-200 families, which in turn target EZH2 and other PRC2 proteins in B-cell lymphomas and prostate cancer [27,28]. Alterations of miRNA expression are observed in GBM and have been linked to the molecular pathogenesis of GBM, as they affect the expression of crucial mRNAs. For instance, several miRNAs abundant in the brain—miR-128, miR-181a, miR-181b, and miR-181c—are downregulated [29], whereas miR-221 is strongly upregulated in GBM [30]. In recent years, miR-9 has become an important miRNA for various cancer types [31,32,33,34]. Moreover, several target genes of miR-9 have been identified, which include E-cadherin, NFκB1, and CXCR4 [35,36,37]. Therefore, understanding the main regulatory mechanism of this malignancy is the key to the development of novel and effective therapeutic strategies for GBM.

CXCR4 is a member of the CXC chemokine receptor subfamily. CXCR4 is one of the most well-studied chemokine systems in tumor growth, metastasis, and angiogenesis. CXCR4 is upregulated in pancreatic cancer, colon cancer, ovarian cancer, lymphoma, medulloblastoma, and glioma, which suggests a critical role for CXCR4 in these cancers [7,38,39,40,41,42]. CXCR4 overexpression has been demonstrated in invasive GBM cells [26], and CXCR4 has been localized in vivo to hypoxic areas in human GBM [43].

The epigenetic function of EZH2 is still unclear in GBM. Here, we investigated the functions and molecular mechanisms of EZH2 and its direct regulation of miR-9 in GBM pathogenesis. Our findings will enhance the understanding the functions of EZH2 and miR-9 and have the potential to provide novel therapeutic approaches for GBM.

## 2. Materials and Methods

### 2.1. Cell Culture

U87-MG and DBTRG-05MG were obtained from the American Type Culture Collection. The GBM8401 GBM cell lines used in the study were purchased from the Bioresource Collection Research Center, Hsinchu, Taiwan. The U87-MG, GBM8401, and DBTRG-05MG human GBM cell lines were cultured in RPMI 1640 medium supplemented with 10% fetal bovine serum (FBS). The cells were maintained in an atmosphere of 5% CO_2_ in a humidified 37 °C incubator.

### 2.2. Comprehensive Analysis of EZH2 and CXCR4 from the Cancer Genome Atlas (TCGA) and the Genotype-Tissue Expression (GTEx) Projects

Gene Expression Profiling Interactive Analysis (GEPIA, http://gepia.cancer-pku.cn/index.html) adopts a standard processing pipeline to analyze the RNA-Seq expression data from GTEx and TCGA, which include 8587 normal and 9736 tumor samples. In the present study, we used GEPIA for tumor/normal differential expression analysis and patient survival analysis regarding EZH2 and CXCR4 expression in GBM.

### 2.3. miRNA Mimic and miRNA Inhibitor

The miR-9 mimic, miRNA-9 inhibitor, and miRNA negative control were purchased from Dharmacon. The miRNA mimic is a double-stranded oligonucleotide designed to mimic the function of the endogenous mature miRNA. miRNA inhibitors are single-stranded, chemically enhanced RNA oligonucleotides designed to bind and sequester the complementary, mature miRNA strand. Cells were transfected with different 10 nM miRNA mimics and inhibitors using RNAiMAX reagent (Invitrogen, Carlsbad, CA, USA), according to the manufacturer’s protocol.

### 2.4. Transient Transfection

Transfections were performed using Lipofectamine 2000 or RNAiMAX (Invitrogen). After 48 h, the transfected cells were used for RNA isolation, total lysate preparation, and migration assays.

### 2.5. Quantitative Reverse Transcriptase–Polymerase Chain Reaction (qRT-PCR)

Total RNA was extracted with TRIzol reagent (Invitrogen). The primers were synthesized by Invitrogen. The qRT-PCR was performed by detecting the hydrolyzed fluorescent probes from the Universal Probe Library (Roche, Pleasanton, CA, USA) using the Light Cycler 480 apparatus (Roche). The specific primer sequences for these genes were as follows: EZH2, forward, 5-GACTGGCGAAGAGCTGTTTT-3 and reverse, 5-TCTTTCGATGCCGACATACTT-3; CXCR4, forward, 5-CCTGCCTGGTATTGTCATCC-3 and reverse, 5-AGGATGACTGTGGTCTTGAGG-3; β-actin, forward, 5-ATTGGCAATGAGCGGTTC-3 and reverse, 5-GGATGCCACAGGACTCCAT-3; hsa-miR-9-RT, 5-GTTGGCTCTGGTGCAGGGTCCGAGGTATTCGCACCAGAGCCAACTCATAC-3; hsa-miR-9, forward, 5-GCGCTCTTTGGTTATCTAGCT-3 and reverse, 5-GTGCAGGGTCCGAGGT-3; RNU6B-RT, 5-GTTGGCTCTGGTGCAGGGTCCGAGGTATTCGCACCAGAGCCAACAAAAATAT-3; RNU6B, forward, 5-TTCCTCCGCAAGGATGACACGC-3 and reverse, 5-GTGCAGGGTCCGAGGT-3. β-actin and RNU6B primers were used as an internal control and to confirm equal loading. The relative quantification of mRNA levels was conducted using the comparative C_t_ method with β-actin or RNU6B as the reference gene and the formula 2^−ΔΔCt^.

### 2.6. Gene Knockdown with Short Hairpin RNAs (shRNAs)

The knockdown of genes was performed with specific shRNAs from the National RNAi Core Facility (Academia Sinica, Taipei, Taiwan) delivered with the lentiviral system according to the instruction manual. The shRNA constructs targeting EZH2 are clones TRCN0000036102 (shEZH2-1) and TRCN0000036103 (shEZH2-2), and the constructs targeting CXCR4 are clones TRCN0000256864 (shCXCR4-1) and TRCN0000256866 (shCXCR4-2). The shRNA construct against luciferase (shLuc), clone TRCN0000072244, was used as a negative control. Cells were cultured until the cell confluency reached ~80%, after which they were infected with lentivirus-bearing specific shRNAs in growth medium containing 8 μg/mL polybrene for 24 h. Then, puromycin was added to the culture medium at a final concentration of 3 μg/mL to allow the selection of infected cells. The efficiency of mRNA downregulation and the specificity for each sequence were evaluated by qRT-PCR.

### 2.7. Transfection of EZH2 and CXCR4 Overexpression Vector

The EZH2 and CXCR4 overexpression plasmid and control vector were transfected into GBM cell lines using Lipofectamine 2000 (Invitrogen) according to the manufacturer’s protocol. The DNA plasmids encoding CXCR4 were from Sino Biological (cat. HG11325-CF). The pcDNA3-His-Myc–EZH2 was a gift from Dr. Mien-Chie Hung.

### 2.8. Chromatin Immunoprecipitation (ChIP) Assay

Chromatin was isolated from GBM cancer cell lines using the ChIP assay kit (Millipore, Inc., Burlington, MA, USA) and precipitated with antibodies against EZH2 (BD Biosciences, San Jose, CA, USA). To determine whether miR-9 was directly regulated by EZH2, we scanned the miR-9 sequence with the UCSC Genome Browser (https://genome.ucsc.edu/), which showed that the miR-9 transcriptional start site (TSS) region is enriched with EZH2 in normal brain astrocytes. Then, immunoprecipitated DNA was PCR-amplified with the three primer sets covering specific regions of the EZH2-associated YY1 binding sites (set1, forward, 5-AGTTGACCCCGATCCAGAC-3 and reverse, 5-CAGAGAAGGGCAGTGGAGAC-3; set2, forward, 5-ACTCCCAAGCGAGTCTCTCA-3 and reverse, 5-ACAACCCTGGGTGATCTCTG-3; set3, forward, 5-CCAGGCCACTTACATCGAG-3 and reverse, 5-GGGAGCTAGGAGGTGAGACC-3). To confirm that DNA was amplified within a linear range, PCR was performed with different numbers of cycles or with a serial dilution of the input DNA, and all the results were shown to fall within this linear range. The PCR products were run on agarose gels and visualized by ethidium bromide staining.

### 2.9. Western Blot Analysis

For total cell lysate extraction, cells were washed twice with ice-cold PBS and lysed in NETN buffer (20 mM Tris at pH 8.0, 150 mM NaCl, 1 mM EDTA at pH 8.0, 0.5% Nonidet P-40) with protease and phosphatase inhibitors (25 mM NaF, 2 mM Na3VO4, 0.1 mM PMSF, 20 μg/mL aprotinin) by sonication. Samples (30 μg) were run on 10% SDS-polyacrylamide gels, and the separated proteins were then blotted onto polyvinylidene difluoride (PVDF) membranes. The primary antibodies used were mouse monoclonal anti-EZH2 (BD Biosciences, 1:1000) and anti-β-actin (Sigma, St. Louis, MO, USA, 1:5000) and rabbit polyclonal anti-CXCR4 (Abcam, Cambridge, UK, 1:1000). The secondary antibodies used were a horseradish peroxidase-conjugated rat anti-mouse secondary antibody (Merck Millipore, Danvers, MA, USA, 1:5000) and an anti-rat secondary antibody (Merck Millipore, Danvers, MA, USA, 1:5000). Immunoreactive bands were visualized with the ECL Detection Reagent (GE, Piscataway, NJ, USA).

### 2.10. Cell Invasion and Migration Assays

The cell invasion assay was conducted using BioCoat Matrigel invasion chambers. Briefly, the Matrigel was added to each chamber to allow the hydration of the Matrigel coating for 1 h immediately before the experiments. Cells (5 × 10^4^) suspended in 100 μL of serum-free medium were then added to the upper chamber of the Matrigel-coated filter inserts. After treatment with surfactin, 700 μL of cultured medium (1:1, *v/v*) with 10% FBS was added to the bottom as a chemoattractant. The chambers were then incubated for 24 h at 37 °C. The migration assay was conducted as described for the invasion assay but without the Matrigel coating. The chambers were then incubated for 24 h at 37 °C. Migrating cells attached to the lower surface of the filter were fixed and stained with 2% ethanol containing 0.2% crystal violet powder. The cells that invaded or migrated through the membrane were then photographed under a light microscope (12.5× and 100×), then lysed with 33% (v/v) acetic acid solution and quantified by absorbance measurement (OD570). The results for migration and invasion were divided by survival to control for the factor of cell proliferation. Thus, the effect of migration and invasion only could be focused on.

### 2.11. Statistical Analysis

The values are expressed as the mean ± SD and were analyzed using an ANOVA with the Bonferroni post hoc test to evaluate differences between multiple groups. All statistical analyses were performed using SPSS for Windows, version 10 (SPSS, Inc., Chicago, IL, USA). A value of *p* < 0.05 was considered to be statistically significant, with three independent experiments performed (mean ± SD, *n* = 3; * *p* < 0.05; ** *p* < 0.01.).

## 3. Results

### 3.1. The Expression Levels of EZH2 and CXCR4 in Cancer Patients Are Correlated with Prognosis

**Figure 1:**
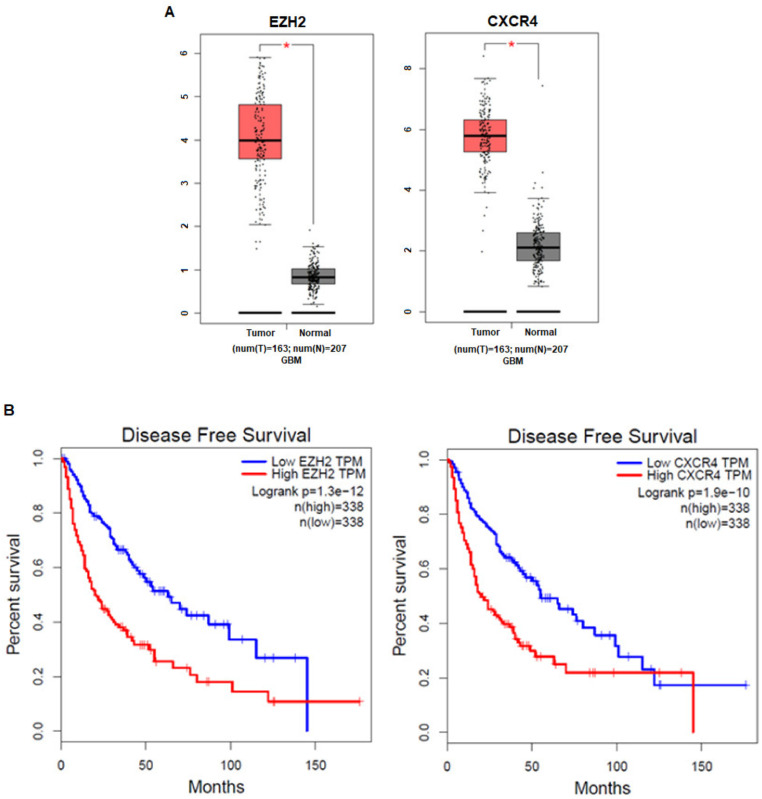
High expression of EZH2 and CXCR4 is associated with the poor prognosis of glioblastoma (GBM) patients. (**A**) EZH2 and CXCR4 mRNA levels are significantly higher in GBM tumor tissues than normal tissues (tumor samples, *n* = 163; normal samples, *n* = 207). * *p* < 0.05: significantly different from normal tissues. (**B**) Both high EZH2 or CXCR4 levels correspond with the reduced disease-free survival of GBM patients based on data obtained from Gene Expression Profiling Interactive Analysis (GEPIA).

To investigate the clinical impact of EZH2 and CXCR4 on GBM cancer progression, we analyzed the relationship between their mRNA expression levels and GBM patient prognosis with GEPIA. We evaluated the association between the EZH2 mRNA level and clinical information or the CXCR4 mRNA level and clinical information (TCGA database). The results revealed that GBM patients with high EZH2 and CXCR4 expression compared to normal people had significantly shorter disease-free survival than those with low EZH2 and CXCR4 expression (Figure 1a,b). These results imply that the enhanced expression of EZH2 and CXCR4 might be involved in the progression of GBM.

### 3.2. EZH2 Maintains CXCR4 Expression in GBM Cell Lines

**Figure 2:**
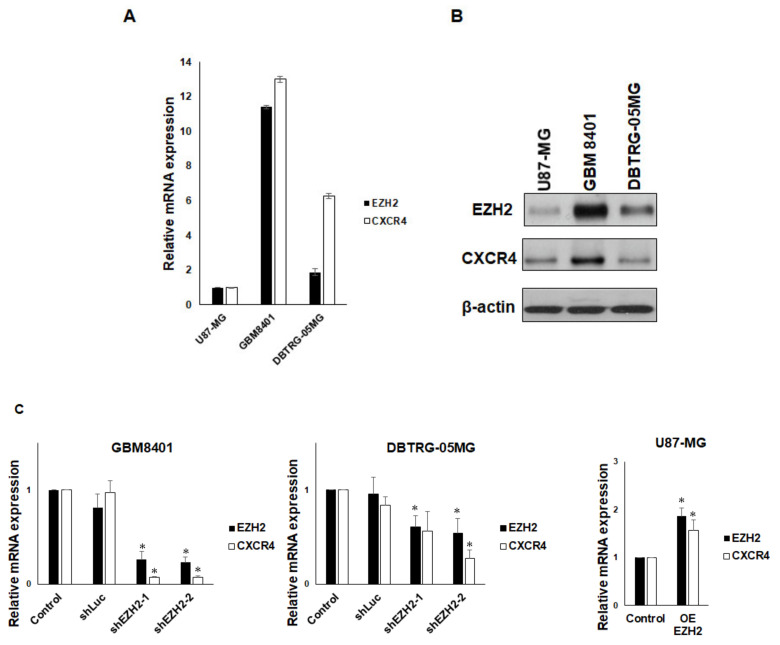

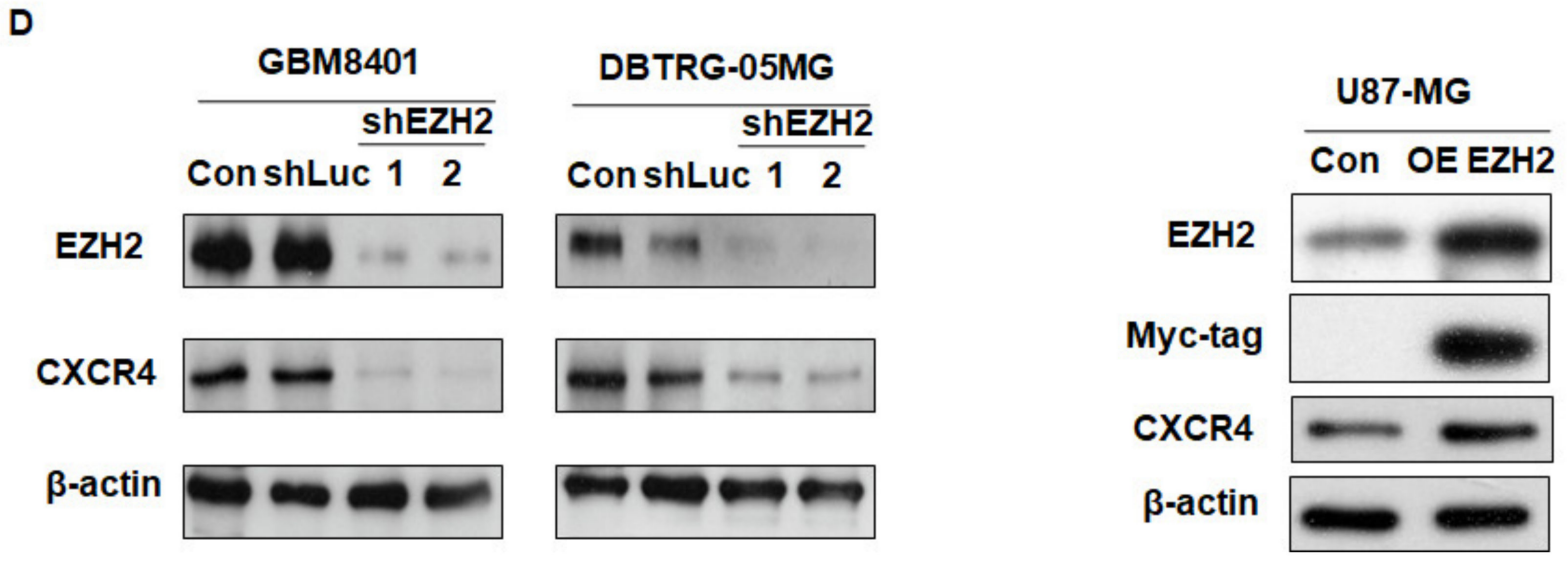
Upregulation of CXCR4 expression by EZH2. (**A**) Differential expression of EZH2 and CXCR4 mRNA in U87-MG, GBM8401, and DBTRG-05MG cells. The expression of EZH2 and CXCR4 was determined using qRT-PCR. (**B**) Western blot analysis of EZH2, CXCR4, and actin (loading control) in whole-cell lysates. (**C**) The effect of shEZH2 or Myc-EZH2 on mRNA expression in GBM8401, DBTRG-05MG, and U87-MG cells was evaluated using qRT-PCR. CXCR4 mRNA expression is shown relative to control. (**D**) Western blot analysis of EZH2 and CXCR4 in EZH2 knockdown/overexpression (OE) GBM cells. (mean ± SD, *n* = 3). * *p* < 0.05. The raw data of Western blots is shown in Figure S1.

To understand the correlation of EZH2 levels with CXCR4 levels in GBM cells, we first examined EZH2 and CXCR4 expression in three human GBM cell lines (U87-MG, GBM8401, and DBTRG-05MG). We detected the lowest levels of EZH2 and CXCR4 mRNA expression in U87-MG cells by qRT-PCR. Conversely, the expression of EZH2 and CXCR4 mRNA was the highest in GBM8401 cells (Figure 2a). Consistent with gene expression, the EZH2 and CXCR4 protein levels were the lowest in U87-MG cells and higher in GBM8401 cells (Figure 2b). These findings indicate that the expression of CXCR4 might correlate with EZH2 expression in GBM cells. To further confirm the relationship of EZH2 and CXCR4 expression in GBM, two different EZH2-specific shRNAs were used to knock down EZH2 expression in GBM cells. Because EZH2 was more highly expressed in GBM8401 and DBTRG-05MG cells, we selected these two GBM cell lines to analyze the correlation between EZH2 expression and CXCR4 expression. We observed that the suppression of EZH2 expression by shRNA was accompanied by a decline in CXCR4 expression in both cell lines, and overexpressed EZH2 also increased the expression of CXCR4 (Figure 2c). Similarly to gene expression, the protein level of EZH2 was reduced/increased in shEZH2-transfected/Myc-EZH2-transfected GBM cells, and the protein level of CXCR4 was also decreased or increased (Figure 2d). These results suggest that EZH2 plays a functional role in the regulation of CXCR4 expression in GBM cell lines.

### 3.3. CXCR4 is a Target of miR-9

**Figure 3:**
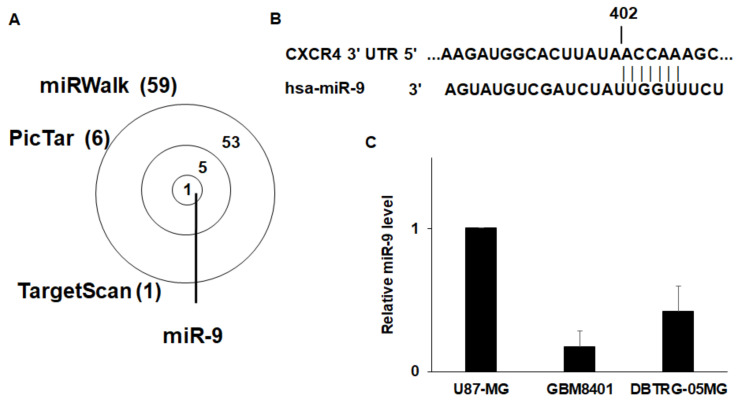

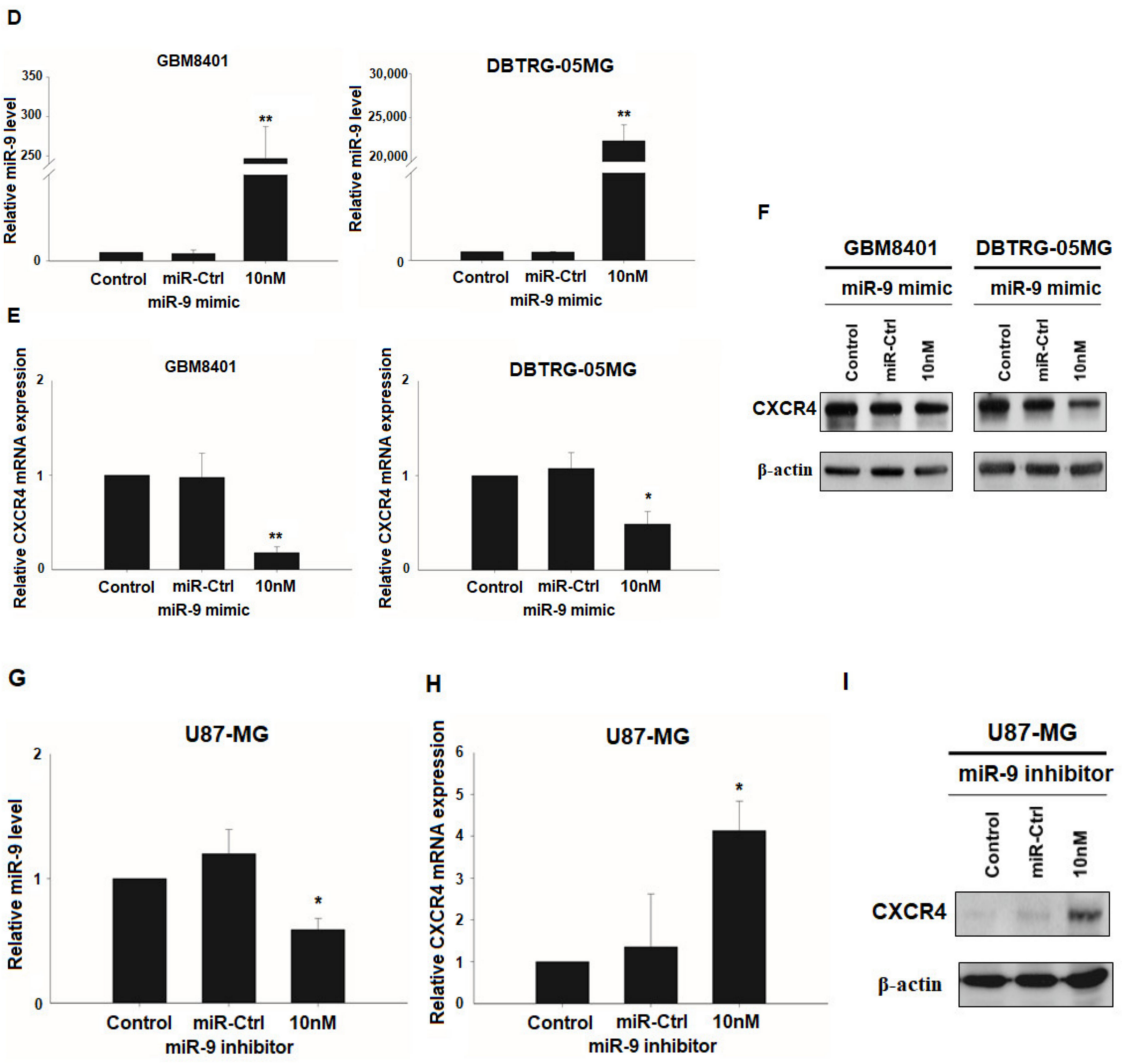
CXCR4 is a target of miR-9. (**A**) The intersection of the analyses using the three algorithms (miRWalk, Pictar, and TargetScan) is shown for the miRNAs that were predicted to target CXCR4 and those that were differentially regulated. (**B**) Binding site of miR-9 predicted in the CXCR4 3′ UTR by TargetScan. (**C**) Differential expression of miR-9 in U87-MG, GBM8401, and DBTRG-05MG cells. The expression of miR-9 was determined using qRT-PCR. (**D**) The effect of the miR-9 mimic (10 nM) in the indicated cells was evaluated using qRT-PCR. (**E**) The relative CXCR4 mRNA levels in GBM8401 and DBTRG-05MG cells 48 h after transfection of the miR-9 mimic (10 nM) or miR-Ctrl (**F**) The protein levels of CXCR4 were measured by Western blotting. β-actin was used as an endogenous control. (**G**) The effect of the miR-9 inhibitor (10 nM) on the miR-9 level in U87-MG cells was evaluated using qRT-PCR. Cells were transfected with miR-9 inhibitor or control, and CXCR4 mRNA (**H**) and protein (**I**) were determined 48 h after transfection. Mean ± SD, *n* = 3. * *p* < 0.05; ** *p* < 0.01. The raw data of Western blots is shown in Figure S2.

miRNAs can regulate cell signaling by interfering with mRNA stability or translation. To identify potential miRNAs involved in EZH2 signaling, we used three computational algorithms—miRWalk, Pictar, and TargetScan—to predict miRNAs that target CXCR4. Interestingly, only miR-9 was identified by all three algorithms (Figure 3a). Furthermore, the analysis indicated that miR-9 can target evolutionarily conserved sequences present in CXCR4 mRNA (Figure 3b) [44]. To further investigate whether miR-9 can regulate CXCR4 expression in GBM cells, we analyzed the miR-9 levels in these three GBM cell lines. miR-9 was highly expressed in U87-MG cells, and the lowest level of mir-9 was found in GBM8401 cells (Figure 3c). miR-9 expression was the opposite of the CXCR4 protein level in the GBM cell lines. We then treated GB8401 and DBTRG-05MG cells with a miR-9 mimic. As shown in Figure 3d, miR-9 was significantly upregulated in both GBM cell lines 48 h after transfection with the miR-9 mimic. Indeed, the CXCR4 mRNA and protein levels both decreased after transfection with the miR-9 mimic (Figure 3e,f), suggesting that the upregulation of miR-9 resulted in a decrease in CXCR4 expression. By contrast, in miR-9 inhibitor-treated U87-MG cells, the miR-9 level decreased (Figure 3g), and the expression of CXCR4’s mRNA and protein was enhanced (Figure 3h,i). These results indicate that miR-9 can regulate CXCR4 protein expression in these GBM cells.

### 3.4. Epigenetic Silencing of miR-9 by EZH2

**Figure 4:**
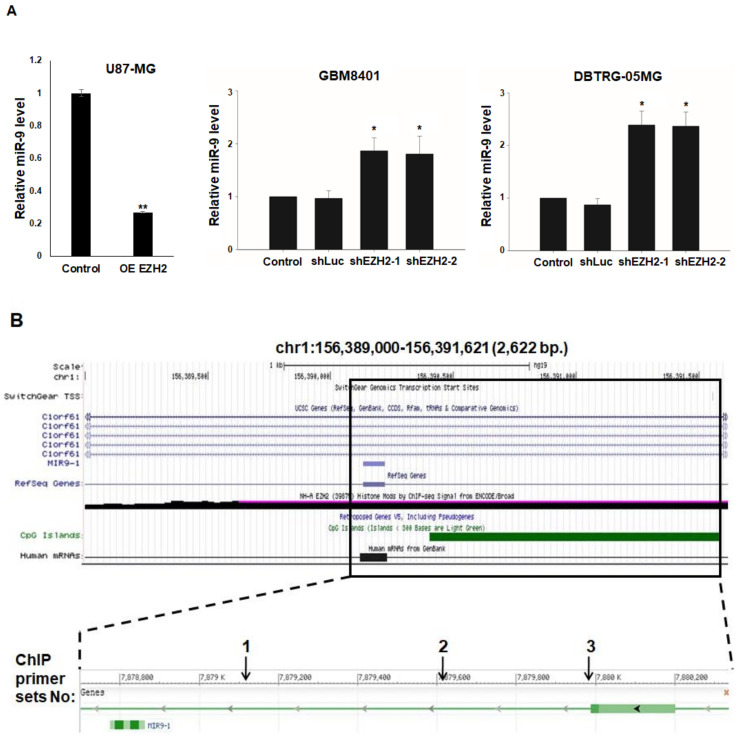

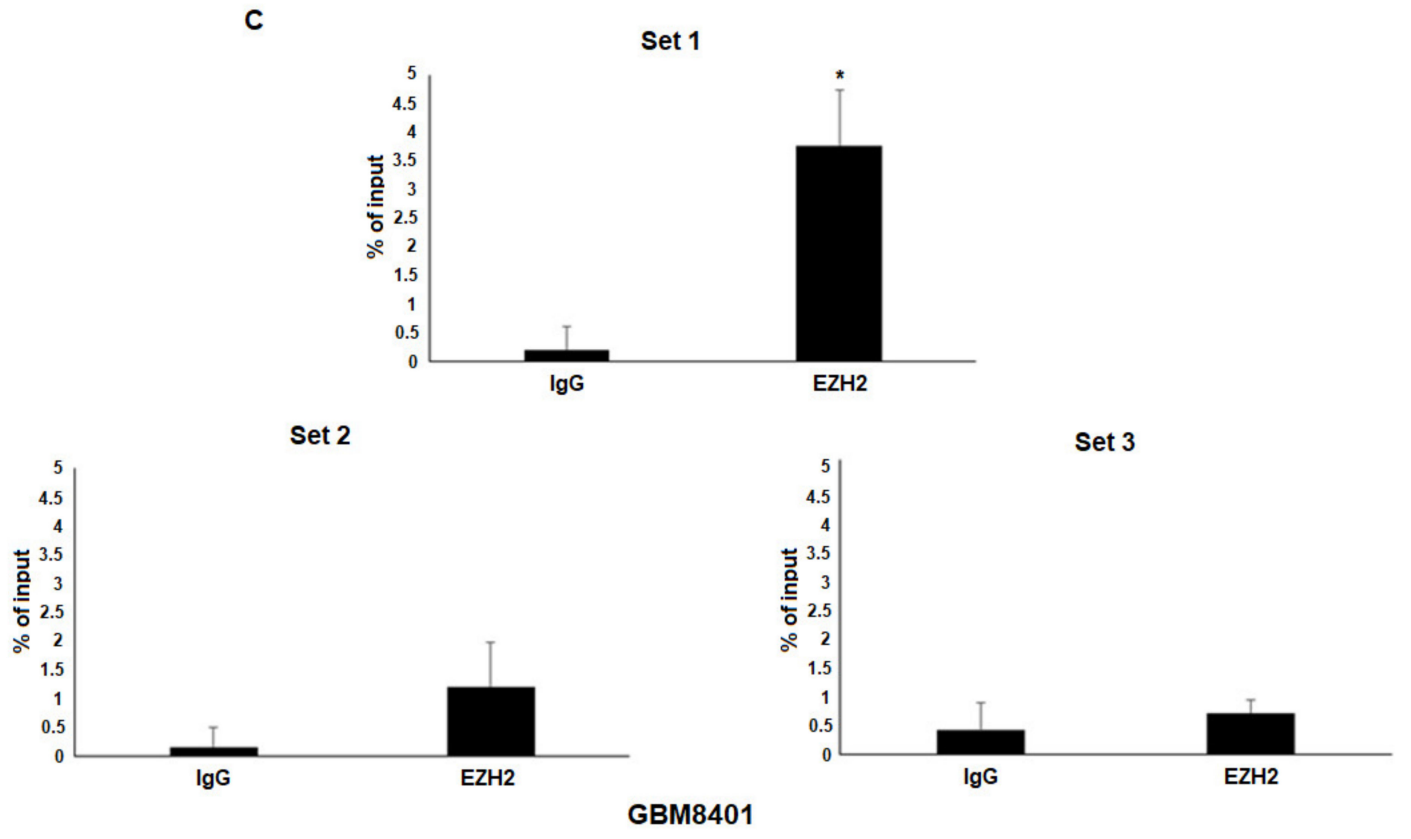
EZH2 represses miR-9 expression. (**A**) Expression of miR-9 in U87-MG, GBM8401, and DBTRG-05MG EZH2 overexpression/knockdown cells was evaluated using qRT-PCR. (**B**) Schematic representation of the miR-9 locus, transcripts, CpG islands, and histone modification (EZH2), which was downloaded and modified from the UCSC Genome Browser. The box frames the genomic section of miR-9 expanded below. Numbered arrows indicated the genomic regions analyzed for EZH2 binding by ChIP assays. (**C**) Chromatin obtained from GBM8401 cells was immunoprecipitated using antibodies for EZH2 or IgG (as a control). Immunoprecipitated DNA was analyzed by PCR using specific primer sets shown in Figure 4b. Mean ± SD, *n* = 3. * *p* < 0.05; ** *p* < 0.01.

To investigate the transcriptional repression of miR-9 by EZH2, we quantitatively determined the miR-9 level in U87-MG, GBM8401, and DBTRG-05MG cells following the overexpression/knockdown of EZH2 expression by Myc-EZH2/shRNA. miR-9 levels were lower/higher in the GBM cells transfected with Myc-EZH2, shEZH2-1, and shEZH2-2 than in those treated with control vector or shLuc (Figure 4a). Thus, miR-9 may be a target of EZH2 for repression in GBM cells. To further study the molecular mechanism of the transcriptional repression of miR-9 by EZH2, we performed ChIP assays in GBM8401 cells using antibodies against EZH2. As mentioned in the introduction, EZH2 is recruited to DNA via the DNA-binding protein YY1. Thus, the EZH2-immunoprecipitated DNA was analyzed by PCR using primer sets designed to amplify regions containing the EZH2-associated YY1 binding sites on the region of miR-9 from 7,879,000 bp upstream to 7,880,000 bp downstream of its TSS (Figure 4b). Under steady-state conditions, ChIP assays indicated that the region around the miR-9 TSS was enriched for EZH2 in GBM8401 cells (Figure 4c). These results suggest that the transcriptional repression of miR-9 occurs through EZH2.

### 3.5. Overexpression of EZH2 or CXCR4 Promotes Cell Migration in GBM Cells

**Figure 5:**
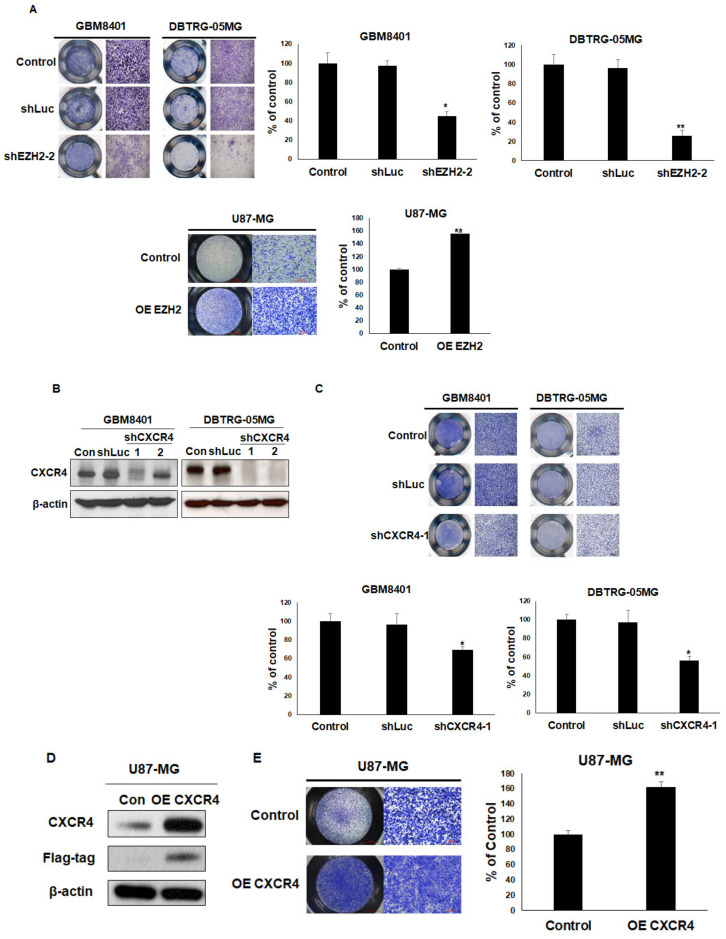
The effects of EZH2 and CXCR4 knockdown or overexpression on GBM cell migration. (**A**) Transwell migration assays were used to evaluate the effects of shEZH2-2 or Myc-EZH2 overexpression on the migration of GBM cells. Representative photographs of transwell assays. The bound crystal violet staining was released with 33% glacial acetic acid and quantified by absorbance measurements (OD570) (**B**) Western blot analysis of CXCR4 protein in CXCR4 knockdown cells. (**C**) Transwell migration assays and quantification of these assays were used to assess the effects of shCXCR4-1 and shLuc (as a control) on GBM cell migration. (**D**) Western blot analysis of CXCR4 protein in Flag-CXCR4 overexpression (OE) U87-MG cells. (**E**) Transwell migration assays and quantification of these assays were used to assess the effects of Flag-CXCR4 on U87-MG migration. Mean ± SD, *n* = 3. * *p* < 0.05; ** *p* < 0.01. The raw data of Western blots is shown in Figure S3.

To investigate whether the level of EZH2 expression was related to cell migration in GBM cells, the expression of EZH2 was upregulated/downregulated by Myc-EZH2/shRNA. After silencing EZH2, a significantly decreased rate of migration was observed in GBM8401 and DTBRG-05MG cells using transwell assays. The overexpression of EZH2 also increased the migration ability of U87-MG (Figure 5a). To understand the role of CXCR4 in GBM cells, we then used shRNAs to silence CXCR4 expression. As shown in Figure 5b, CXCR4 was dramatically knocked down in these GBM cells, which resulted in reduced cell migration in the transwell assays (Figure 5c). Furthermore, the overexpression of Flag-CXCR4 also enhanced cell migration in U87-MG in the transwell assays (Figure 5d,e). These results suggest vital roles for EZH2 and CXCR4 in mediating the migration of GBM cells.

### 3.6. miR-9 Significantly Regulates the Invasion Ability of GBM Cells

**Figure 6:**
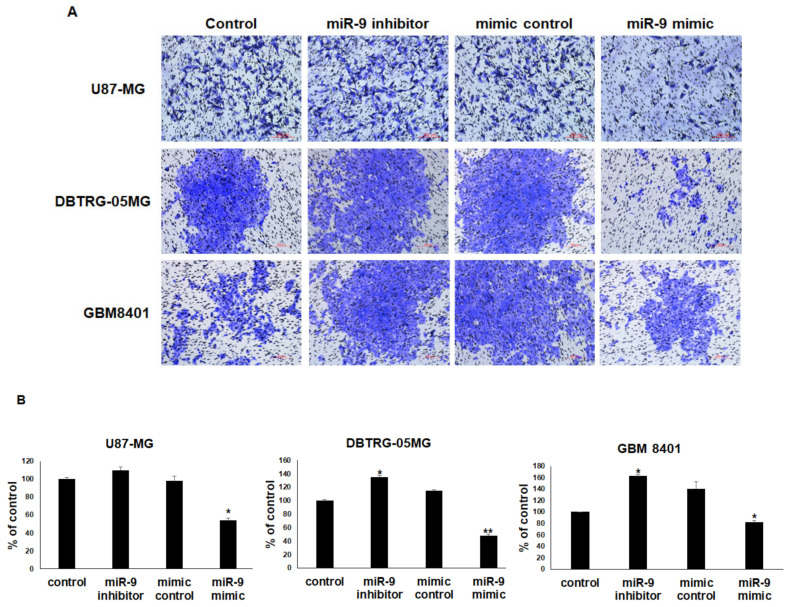
miR-9 significantly regulates the invasion ability of GBM cells. (**A**) Transwell invasion assays were used to evaluate the effects of miR-9 on the invasion of GBM cells. Representative photographs of transwell invasion assays. (**B**) The bound crystal violet staining was released with 33% glacial acetic acid and quantified by absorbance measurements. Mean ± SD, *n* = 3. * *p* < 0.05; ** *p* < 0.01.

The finding that CXCR4 mRNA was significantly decreased 48 h after the transfection of GBM cells with miR-9 suggested that the upregulation of miR-9 resulted in a decrease in CXCR4 expression. Therefore, U87-MG, GBM8401, and DBTRG-05MG cells were treated with miR-9 inhibitor or miR-9 mimic to evaluate the effect on invasion ability with Matrigel invasion assays. The inhibition of miR-9 significantly enhanced GBM8401 and DTBRG-05MG invasion, whereas the overexpression of miR-9 reduced all GBM cells’ invasion (Figure 6A,B). These results indicate that miR-9 can regulate the invasion ability of GBM cells.

## 4. Discussion

GBMs are the main primary central nervous system tumors in humans, accounting for 70% of all malignant primary brain tumors. Thus, human gliomas have prompted many studies focused on their genetic variation and molecular expression patterns in an effort to characterize different tumor subgroups; to understand the malignant behavior of these tumors; and to identify valuable, reliable molecular targets for future targeted therapies. In the current study, we demonstrated that glioma patients expressed aberrant *EZH2* and *CXCR4* levels and that these levels were significantly associated with patient survival according to GEPIA. These strong associations suggest that EZH2 and CXCR4 overexpression promotes tumor progression and that EZH2 and CXCR4 could possibly be used as biomarkers for a more aggressive phenotype of gliomas. To the best of our knowledge, this is the first study to demonstrate the effect of EZH2–miR-9–CXCR4 axis regulation.

Promoter DNA methylation is the most common epigenetic modification associated with gene silencing in cancer. As a crucial epigenetic regulator of gene expression, EZH2 is functionally involved in many aspects of multiple carcinomas, such as tumor initiation, tumor cell survival, chemoresistance, invasiveness, metastasis, and angiogenesis [45]. EZH2, as a part of the PRC2 complex, appears to recruit DNA methyltransferases to its target genes, resulting in the methylation of adjacent CpG islands and subsequent gene silencing [46]. EZH2-mediated H3K27 methylation may be a prerequisite for promoter DNA methylation. The downregulation of miRNAs in some tumors may be mediated by CpG island hypermethylation [47].

The sequence-specific DNA binding protein YY1 interacts with EED and recruits PRC2 to a specific chromatin domain to be repressed [48]. Our results support this idea, as we observed that miR-9 was silenced through EZH2 expression. Our result of decreased miR-9 expression in GBM cell lines is consistent with a previous report [49]. miR-9 is deregulated in several other types of cancer, including prostate cancer [10], bladder transitional cell carcinoma [50], and gastric cancer [51], and is strongly correlated with migration, invasion, and metastasis [10,51]. We found that EZH2 epigenetically silenced miR-9, which directly targets the oncogenic signaling of CXCR4 in glioblastoma cell lines. Furthermore, in GBM8401 and DBTRG-05MG cells treated with the miR-9 inhibitor, invasion was enhanced, and in GBM cells treated with the miR-9 mimic, invasion was reduced. Thus, miR-9 may play important roles in these GBM cells.

CXCR4 has been implicated in tumor metastasis to distant organs via blood vessels in several tumor models, including melanoma metastasis to the lung, prostate cancer metastasis to the bone, and neuroblastoma metastasis to the bone marrow [52]. However, some research has indicated that the EZH2-mediated loss of miR-622 determines CXCR4 activation in hepatocellular carcinoma [53]. However, miR-622 also downregulates K-Ras, and its low expression was associated with a worse prognosis [54]. The impaired translational repression of CXCR4 by miR-9 causes EZH2 overexpression in GBM cell lines. In this study, we identified CXCR4 as a target of miR-9 and demonstrated that miR-9 downregulated the mRNA and protein levels of CXCR4 in GBM. This result is consistent with recent reports [44,55]. Here, we demonstrated a role for CXCR4 overexpression in GBM, which could be inhibited by miR-9 or the knockdown of EZH2. We identified roles for EZH2, miR-9, and CXCR4 in cellular migration, indicating a pro-tumoral function in GBM.

## 5. Conclusions

**Figure 7:**
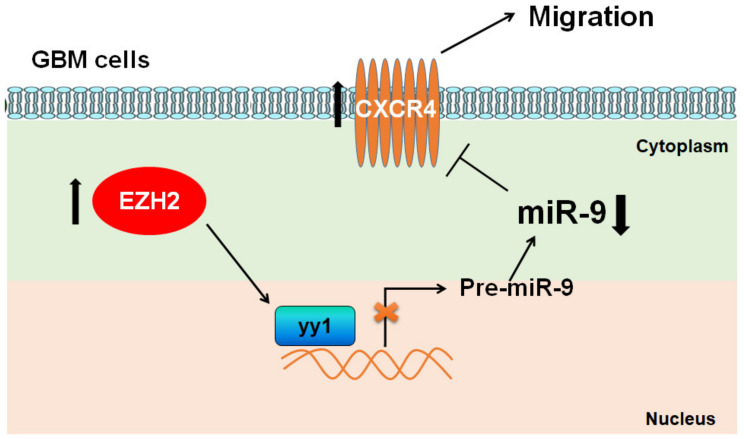
Proposed signaling for EZH2/miR-9/CXCR4 modulation of GBM cell migration and invasion. EZH2 modulates the expression of miR-9, which directly targets the oncogenic signaling of CXCR4 in GBM cell lines.

In conclusion, our results provide new insights into the EZH2/miR-9/CXCR4 pathway, which promotes GBM cell migration (Figure 7). Functional analyses identified miR-9 as a potential tumor suppressor in the development and progression of GBM through the targeting of the key metastasis promoter, CXCR4. Our results provide evidence for EZH2 and miR-9 as potential metastatic markers and therapeutic targets in the treatment of GBM.

## Supplementary Materials

The following are available online at https://www.mdpi.com/2072-6694/12/7/1781/s1, Figure S1: Raw data of Western blots from Figure 2, Figure S2: Raw data of Western blots from Figure 3, Figure S3: Raw data of Western blots from Figure 5.
