# Supplementary material for: Epigenetic Silencing of miR-9 Promotes Migration and Invasion by EZH2 in Glioblastoma Cells
Source: Cancers (Basel). 2020 Jul 3;12(7):1781. doi: 10.3390/cancers12071781 (PMC7408254; doi:10.3390/cancers12071781)
Supplement: Supplementary file 1 [file cancers-12-01781-s001.pdf]

Supplementary Materials

Epigenetic Silencing of miR-9 Promotes Migration and Invasion by EZH2 in Glioblastoma Cells

Yi-Chung Chien, Jia-Ni Chen, Ya-Huey Chen, Ruey-Hwang Chou, Han-Chung Lee and Yung-Luen Yu

A

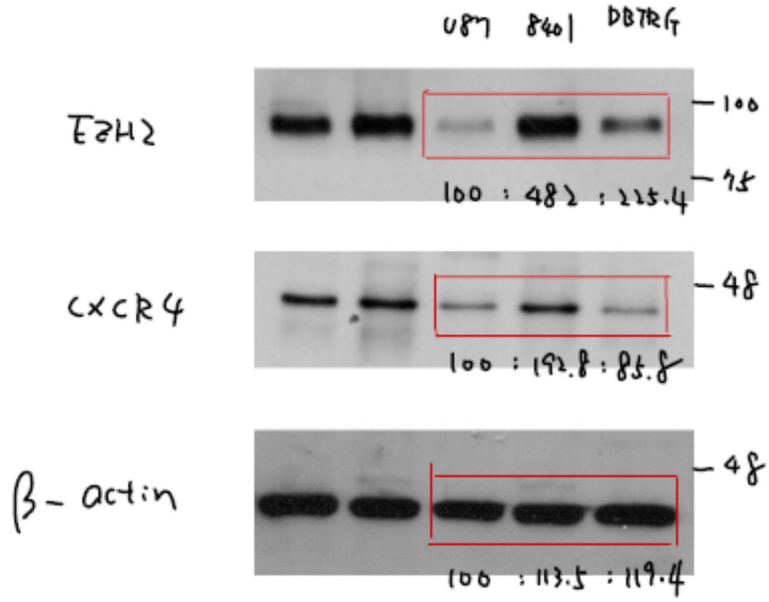

B

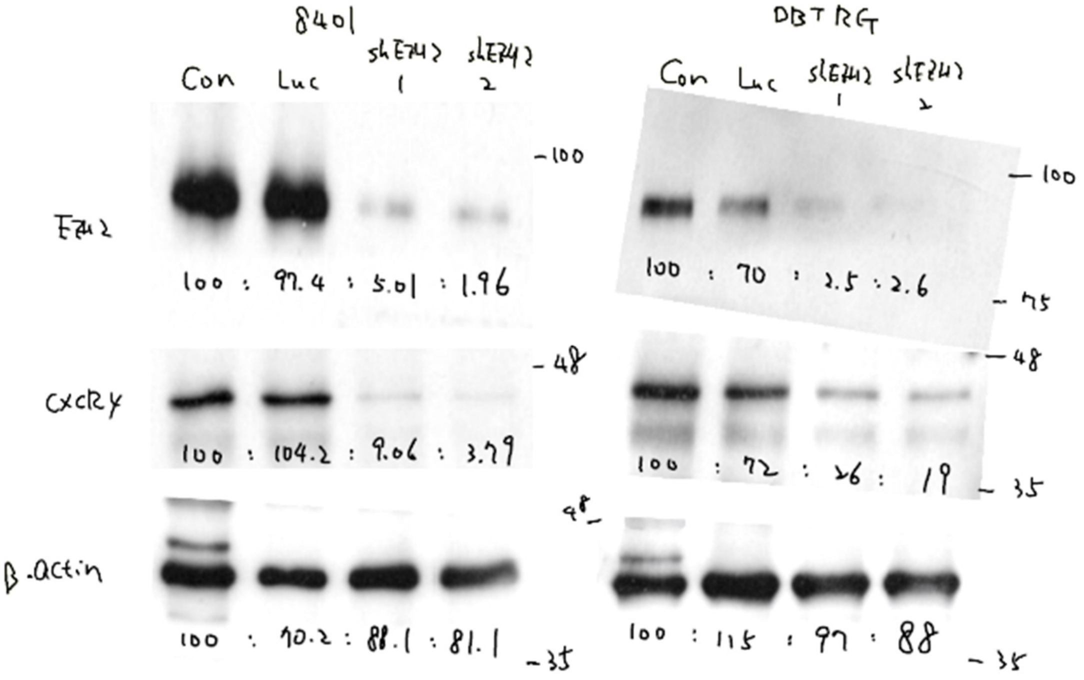

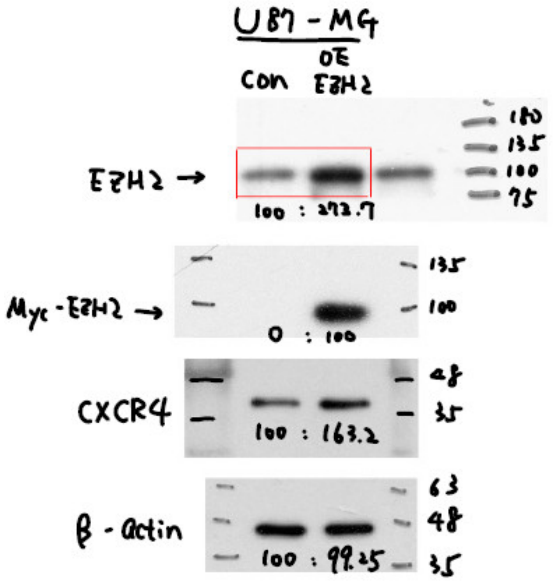

Figure S1. Raw data of Western blots from Figure 2. (**A**) Raw data from Figure 2B. (**B**) Raw data from Figure 2D.

A

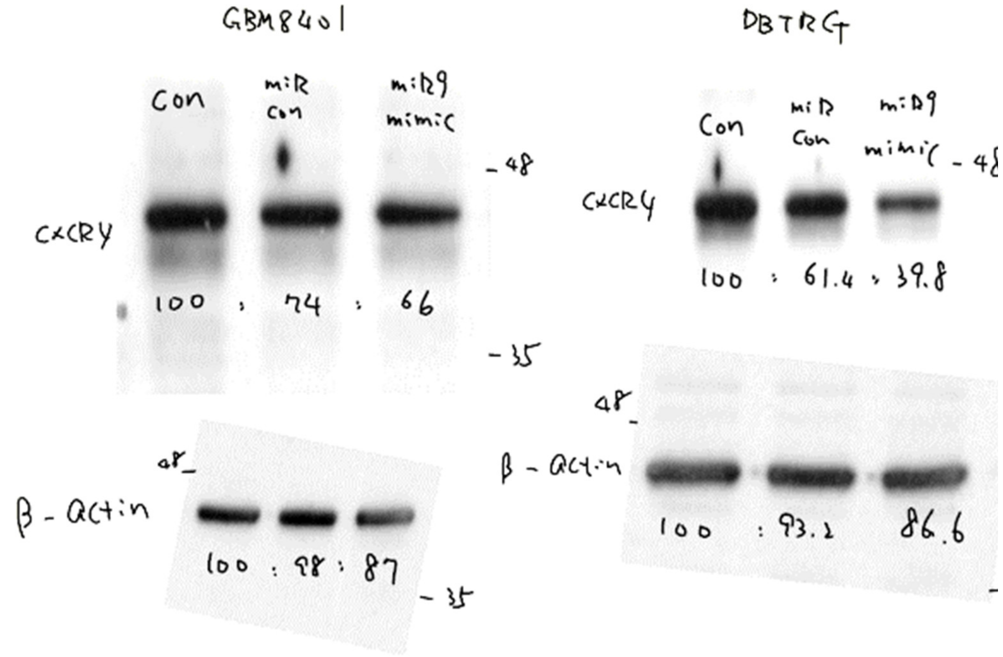

B

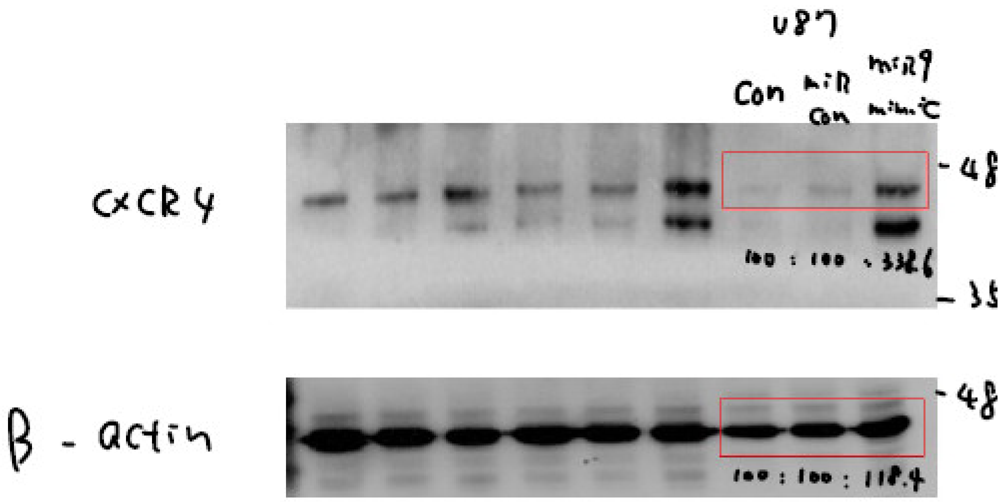

Figure S2. Raw data of Western blots from Figure 3. (**A**) Raw data from Figure 3F. (**B**) Raw data from Figure 3I.

A

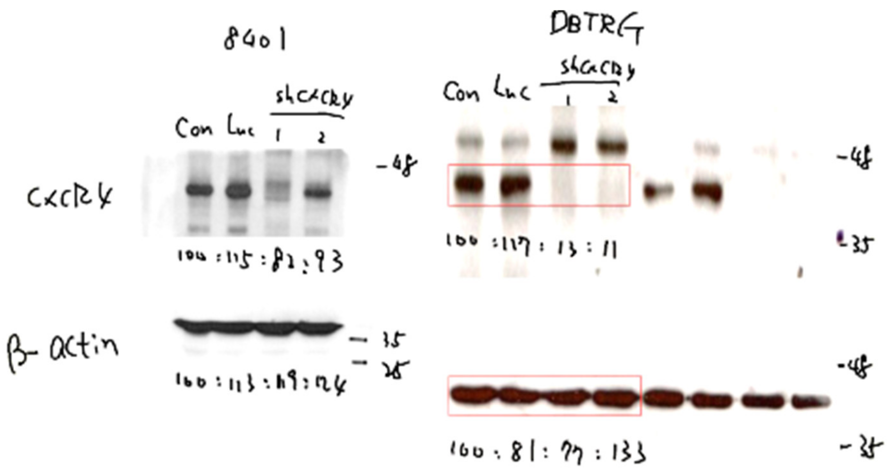

B

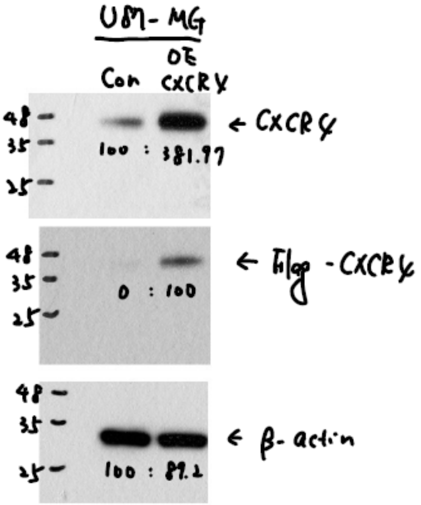

Figure S3. Raw data of Western blots from Figure 5. (**A**) Raw data from Figure 5B. (**B**) Raw data from Figure 5D.

© 2020 by the authors. Licensee MDPI, Basel, Switzerland. This article is an open access article distributed under the terms and conditions of the Creative Commons Attribution (CC BY) license (http://creativecommons.org/licenses/by/4.0/).
